# Short-Term Pre-Operative Protein Caloric Restriction in Elective Vascular Surgery Patients: A Randomized Clinical Trial

**DOI:** 10.3390/nu13114024

**Published:** 2021-11-11

**Authors:** Peter Kip, Thijs J. Sluiter, Jodene K. Moore, Abby Hart, Jack Ruske, James O’Leary, Jonathan Jung, Ming Tao, Michael R. MacArthur, Patrick Heindel, Alwin de Jong, Margreet R. de Vries, M. Furkan Burak, Sarah J. Mitchell, James R. Mitchell, C. Keith Ozaki

**Affiliations:** 1 Department of Surgery and the Heart and Vascular Center, Brigham & Women’s Hospital and Harvard Medical School, 75 Francis Street, Boston, MA 02115, USA; pkip@alrijne.nl (P.K.); T.J.Sluiter@lumc.nl (T.J.S.); ahart9@bwh.harvard.edu (A.H.); jwr95@cornell.edu (J.R.); jamesjosepholeary4@gmail.com (J.O.); mtao2@partners.org (M.T.); PHEINDEL@PARTNERS.ORG (P.H.); mfburak@hsph.harvard.edu (M.F.B.); 2 Department of Molecular Metabolism, Harvard T.H. Chan School of Public Health, Boston, MA 02115, USA; jonjung@gmail.com (J.J.); michaelrobert.macarthur@hest.ethz.ch (M.R.M.); sarahjayne.mitchell@hest.ethz.ch (S.J.M.); peterkip.pk@gmail.com (J.R.M.); 3 Einthoven Laboratory for Experimental Vascular Medicine, Department of Surgery, Leiden University Medical Center, 2333 ZC Leiden, The Netherlands; A.de_Jong.HLK@lumc.nl (A.d.J.); m.r.de_vries@lumc.nl (M.R.d.V.); 4 Department of Systems Biology, Harvard Medical School, Boston, MA 02115, USA; Jodene_Moore@hms.harvard.edu; 5 School of Medicine, University of Glasgow, Glasgow G12 8QF, UK; 6 Department of Health Sciences and Technology, ETH Zurich, 8092 Zurich, Switzerland

**Keywords:** dietary restriction, vascular surgery, metabolic fitness

## Abstract

(1) Background: Vascular surgery operations are hampered by high failure rates and frequent occurrence of peri-operative cardiovascular complications. In pre-clinical studies, pre-operative restriction of proteins and/or calories (PCR) has been shown to limit ischemia-reperfusion damage, slow intimal hyperplasia, and improve metabolic fitness. However, whether these dietary regimens are feasible and safe in the vascular surgery patient population remains unknown. (2) Methods: We performed a randomized controlled trial in patients scheduled for any elective open vascular procedure. Participants were randomized in a 3:2 ratio to either four days of outpatient pre-operative PCR (30% calorie, 70% protein restriction) or their regular ad-libitum diet. Blood was drawn at baseline, pre-operative, and post-operative day 1 timepoints. A leukocyte subset flow cytometry panel was performed at these timepoints. Subcutaneous/perivascular adipose tissue was sampled and analyzed. Follow-up was one year post-op. (3) Results: 19 patients were enrolled, of whom 11 completed the study. No diet-related reasons for non-completion were reported, and there was no intervention group crossover. The PCR diet induced weight loss and BMI decrease without malnutrition. Insulin sensitivity was improved after four days of PCR (*p* = 0.05). Between diet groups, there were similar rates of re-intervention, wound infection, and cardiovascular complications. Leukocyte populations were maintained after four days of PCR. (4) Conclusions: Pre-operative PCR is safe and feasible in elective vascular surgery patients.

## 1. Introduction

Vascular surgery patients suffer from peri-operative complications, such as myocardial infarction, ischemic stroke [1], and infections [2], at rates higher than those in many other surgical populations. Additionally, numerous procedure-specific challenges exist in vascular surgery. For example, in patients undergoing revascularization for peripheral artery disease (PAD), loss of graft patency is a constant threat. Over 200 million individuals worldwide suffer from PAD [3]. In the past decade, the number of lower extremity interventions for PAD has nearly doubled [4], and revascularization procedures are a high-volume mainstay in treating arterial occlusive disease [5]. Unfortunately, interventions for PAD are associated with high failure rates [6,7], resulting in frequent re-admission [8] and often requiring re-interventions [6,7]. Such complications can lead to enormous patient suffering and even death, ultimately resulting in an immense economic and social burden [9]. Therefore, new strategies to improve peri- and post-op complication rates following vascular surgery are urgently needed.

Some progress in treating vascular surgery patients has been made by implementing Enhanced Recovery After Surgery (ERAS) protocols [10], which have been demonstrated to reduce post-operative inpatient length of stay [11]. Key parts of these protocols focus on optimizing nutrition in the peri- and postoperative time period together with early patient mobilization, but guidelines make few recommendations concerning optimal pre-operative nutrition. Interestingly, precise requirements for “optimal” pre-operative nutrition are not globally agreed upon, and recent evidence has emerged that a more extensive dietary preconditioning strategy, such as protein restriction, during the days to weeks leading up to the procedure could potentially profoundly impact post-operative outcomes [12]. This concept of dietary restriction (DR), i.e., restriction of calories, proteins, specific amino-acids, or a combination of the aforementioned—but without malnutrition, is best known for its ability to increase health and lifespan in various species when applied long-term (months to years) [13].

Various forms of DR, including caloric and protein restriction, can modulate the physiologic stress response to surgical injury, which likely underlies peri-operative complications and contributes to the limited lifespan of some vascular reconstructions. Short-term DR, with durations spanning three days to four weeks, has been demonstrated to enhance recovery of renal function [14,15,16] and mitigate hepatic damage [14,16,17,18] after surgical ischemia-reperfusion injury. Surgical stress signaling in soft tissues, such as adipose [19], is also attenuated by DR [20]. Importantly, short-term DR does not appear to impair wound healing [21]. In vascular surgery models, DR attenuates arterial intimal hyperplasia [15], improves vein graft remodeling after rodent bypass surgery [22], and stimulates angiogenesis after femoral artery ligation [23]. Interestingly, combining two forms of DR (i.e., protein and caloric restriction (PCR)) appears more efficacious in yielding protection after surgical stress than unilateral restriction of calories or proteins [24]. Mechanistically, one of the mechanisms through which DR confers its benefits in various surgical models can be explained by upregulation of endogenous hydrogen sulfide (H_2_S) [16], a gaseous vasodilator [25] with broad anti-inflammatory [26], antioxidant [27], and anti-atherosclerotic [28,29] properties.

In a prior pilot study, patients undergoing elective carotid endarterectomy [30] underwent a pre-operative PCR intervention while being closely monitored as inpatients for three days prior to scheduled surgery to assess the safety of this specific diet in this fragile population. All patients completed the study without experiencing any adverse events or dietary compliance issues. In the current study, we focused on evaluating the feasibility of a pre-operative PCR diet in the vascular surgery patient population. To test this, we optimized the study design to be more translationally relevant by performing it in an outpatient population and including all open vascular surgery operations. The current study assesses the feasibility of and compliance with a short-term PCR diet in a cross-section of vascular surgery patients. Additionally, we investigated the effects of a pre-operative PCR diet on clinical parameters and metabolic health. Furthermore, we interrogated the innate and adaptive immune response to the diet and the surgical intervention, specifically evaluating the effects of the diet on both the response to surgical stress and endogenous H_2_S modulation in these immune cell subsets.

## 2. Material and Methods

### 2.1. Trial Design and Setting

**Figure 1:**
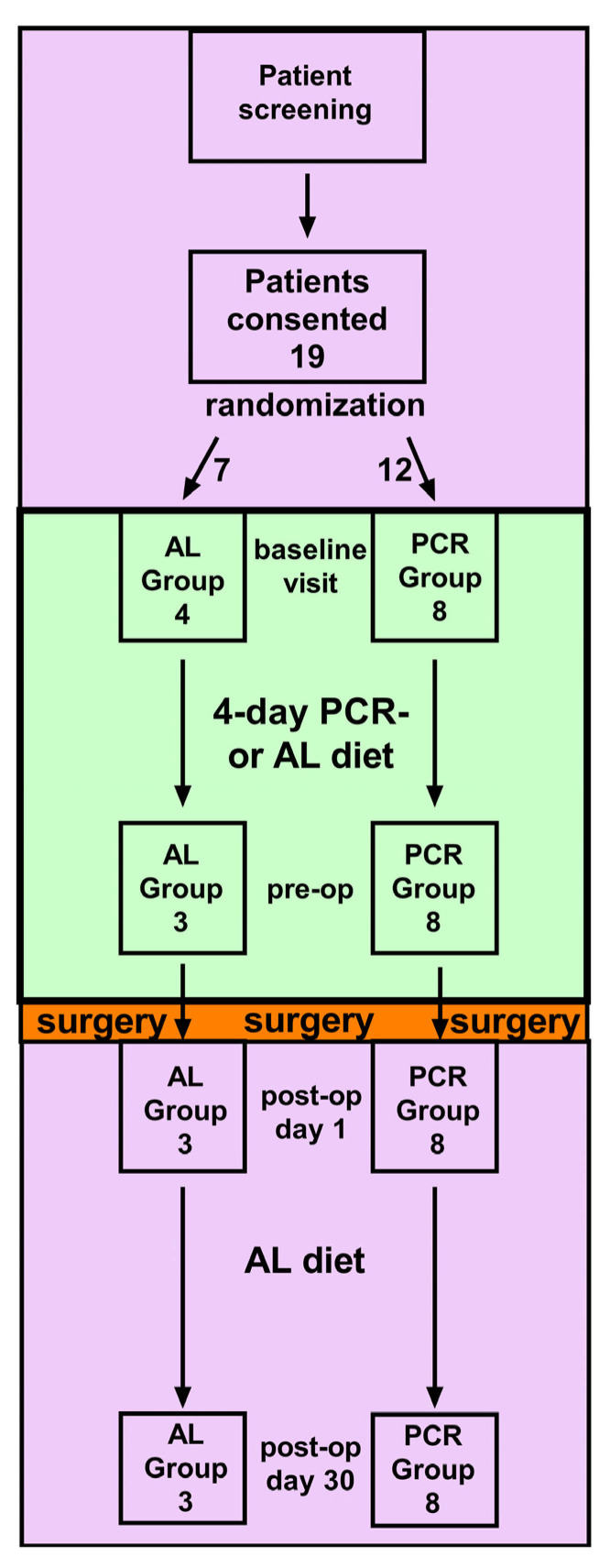
Study design. After consent, patients were randomized into ad-libitum (AL) or protein calorie restriction (PCR) groups. Patient characteristics, clinical parameters, and blood were collected at the baseline visit, pre-operative visit, and at post-op day 1 (POD1). Perioperatively, we collected perivascular (PVAT) and subcutaneous (SQ) adipose tissue. Prospective follow-up was conducted until POD30.

This randomized controlled outpatient clinical trial was approved by the Partners Human Research Committee institutional review board and registered with ClinicalTrials.gov (Identifier: NCT04013412) to enroll subjects at one academic tertiary medical center (Brigham and Women’s Hospital, Boston, MA, USA). Study subjects were recruited from a cohort of patients who were scheduled to undergo an elective open vascular surgery procedure (defined below) at a single institution. After initial screening and patients were deemed eligible, written informed consent was obtained by a physician-investigator from subjects before enrollment. Patients were then randomized at their baseline visit (scheduled between 30 and 5 days pre-surgery) to either the ad-libitum (AL) group or a four-day pre-operative protein-caloric restriction (PCR) diet to be consumed in an out-patient setting. Patients continued their assigned diets for four days leading up to the scheduled surgery until midnight at the day of surgery, when both cohorts were instructed NPO (nil per os) except selected medications. On the day of surgery, patients underwent typical same-day admission processes and received postoperative care per standard clinical practice. Immediately post-op, all patients were advanced rapidly to an ad-libitum diet as tolerated, and patients were followed prospectively until post-op day 30 (Figure 1, study design). This randomized controlled clinical trial took place from May 2019 until February 2020, when enrollment was terminated due to the global COVID-19 pandemic.

### 2.2. Inclusion and Exclusion Criteria

#### 2.2.1. Inclusion Criteria

Patients eligible for inclusion included all patients greater than 18 years old who presented for one of the following elective procedures: carotid artery endarterectomy, aortic/iliac aneurysm repair (open or endovascular only if groin cut down planned), open lower extremity arterial procedures (bypasses, aneurysm repair, arterial and bypass graft reconstructions), major amputations of the lower extremity (below knee and above knee amputations), and open hemodialysis access procedures.

#### 2.2.2. Exclusion Criteria

Patients were excluded from our study for intolerance or allergy to any of the ingredients in the PCR diet, active infection, pregnancy, malnutrition (serum albumin < 3 g/dL), uncontrolled diabetes (HgbA1c > 12%), substance dependency that could interfere with protocol adherence and assent as determined by the principal investigator, active non-cutaneous cancer under treatment with chemotherapeutics or radiation, emergency surgery, active participation in any other interventional or randomized study, and participation in the current study within the past 30 days.

### 2.3. Randomization and Intervention

After written informed consent was obtained, randomization was performed in blocks via a 3:2 ratio, and treatment allocation was random via https://www.randomizer.org (accessed on 1 April 2020). We opted for a 3:2 randomization design to rigorously study diet compliance and safety in the PCR group while maintaining sufficient numbers of AL patients for controls. When randomized to the PCR group (Scandishake (any of four flavors: vanilla, strawberry, banana cream, or caramel) mixed with almond milk), patients received their PCR diet for the next four days, which was calculated individually (see “Dietary Compliance”) as a total daily volume to achieve 30% caloric restriction and 70% protein restriction based on body weight and activity level. For the macronutrient ratio of Scandishake in Kcal and percentage, see Table 1.

When allocated to the AL group, patients could continue their ad-libitum diet.

### 2.4. Blinding

All non-essential clinical staff were blinded to the study arm, including basic science study staff, to ensure unbiased interpretation of clinically obtained data and specimens. All experiments and assays on blood and tissue samples were performed blinded, and study staff was unblinded after trial, and specimen analysis was completed.

### 2.5. Dietary Compliance

Patients enrolled in the PCR group received 16 portions of the PCR Scandishake diet at their baseline visit to accommodate for a four-day outpatient PCR diet. For macronutrient ratio of the Scandishake diet, see Table 1. To calculate the volume of PCR diet needed in order to achieve a 30% caloric and 70% protein restriction, qualified dieticians employed the Mifflin St. Jeor equation [31] based on gender, age, height, weight, and activity factor and combined this with the patients’ last meal recall. To monitor diet compliance in the PCR group and food intake in the AL group, a MealLogger app was utilized for study subjects to self-record their intake. The PCR group was encouraged to monitor anytime they deviated from the PCR diet.

### 2.6. Clinical Parameters

At the baseline visit, immediately pre-op, post-op day 1 (POD1), and POD30, study participants’ height, weight (utilizing the same scale for all patients/timepoints), temperature, blood pressure, and heart rate was recorded. Clinically important perioperative outcomes, including mortality (all cause and cardiovascular), stroke, myocardial infarction, coronary revascularization, thrombotic complications, and reinterventions related to the index case, were determined through review of the electronic health record.

### 2.7. Blood Draws

Fasting blood draws were performed at the baseline clinic visits, immediately pre-op on POD0, and on POD1 and collected in designated tubes. At each timepoint, 5.0 mL of whole blood was collected in an ethylenediaminetetraacetic acid (EDTA)-covered tube for a complete blood count with differential, and 10 mL of whole blood was collected in a red top tube (RTT) for a basic metabolic panel combined with cortisol, insulin growth factor-1 (IGF-1), and c-reactive protein (CRP). Baseline and pre-op testing also included pre-albumin. These tests were all performed by technicians at the Center for Clinical Investigation at Brigham and Women’s Hospital and tested by the laboratory Corporation of American Holdings (LabCorp). A second RTT with 8.0 mL of whole blood was collected. After incubation for 30 min on wet ice, the tube was centrifuged (Thermo Forma 5681 3L GP) at 2500× *g* for 15′ at 4 °C. Supernatant was then collected in several 1.5-mL Eppendorf tubes and stored as serum at −80 °C until further analysis. At each timepoint, a second EDTA tube with 8.0 mL of whole blood was also collected at room temperature (RT) and immediately processed for flow cytometry analysis as described below.

### 2.8. Adipose Tissue Biopsy

At the start of the surgical procedure, immediately following the first incision, a subcutaneous adipose tissue sample of approximately 1 cm^3^ was collected in a 1.5-mL Eppendorf tube and flash frozen in liquid nitrogen. Next, when the surgical field around the target vessel was explored, approximately 1 cm^3^ of arteriovenous perivascular adipose tissue was collected in a 2.0 mL Cryotube and flash frozen in liquid nitrogen. Both samples were then stored at −80 °C until further processing for Luminex analysis.

### 2.9. Flow Cytometry Panel Creation & Validation

To characterize lymphocyte and monocyte subsets in the blood of study participants before and after diet and surgery, a comprehensive antibody panel and processing and gating strategy was developed based on a previously published panel [32]. First, antibodies (listed below) were titrated on healthy donor human peripheral blood mononuclear cells (PBMC) to determine the optimal staining index, resulting in the following antibody cocktail described in Table 2.

This panel was combined with a fluorescent probe that binds free H_2_S [33]: P3 (EMD Millipore, cat#534329, 378/524 nm [Ex/Em]), and a Zombie Violet Fixable Viability live/dead dye to exclude dead cells (Biolegend, cat#423113, 400/423 nm [Ex/Em]).

For single-compensation control of non-abundant epitopes (i.e., CD25, CD38, CD56, CD127, CD183, CD196), the following CD4 antibodies were used as described in Table 3.

Fluorescence minus one (FMO) control were employed for the following epitopes: CD25, CD38, CD56, CD127, CD183, and CD196. An FMO was also run for P3. To account for inter-patient and inter-timepoint variability, healthy donor PBMCs were cryopreserved and thawed (see below) during patient blood processing to employ as single-compensation FMO controls.

### 2.10. Isolation of Control Peripheral Blood Mononuclear Cells from Healthy Donors

We isolated PBMCs from healthy donors and cryopreserved these cells to titrate and validate our antibody panel as well as to use in our single control and FMO compensation assays. Approximately 250 mL of healthy donor whole blood in EDTA tubes was acquired from Research Blood Products LLC Boston, delivered at RT. Upon delivery, whole blood was diluted 1:1 in 2% heat-inactivated fetal bovine serum (Hi-FBS) (Sigma, Saint-Louis, MO, USA, cat#F4135) in Hanks’ Balanced Salt Solution (HBSS), without calcium or magnesium (Thermo Fisher, Waltham, MA, USA, cat#14170112), i.e., washing buffer (WB). After careful mixing, the suspension was layered on top of an equal volume of Lymphoprep density gradient medium (Stem Cell, Vancouver, BC, Canada, cat#07811) in a 50-mL tube and centrifuged at 800× *g* for 20′ at RT with brakes off. Afterwards, using a sterile dropper pipet, the PBMC layer was collected in a new 50-mL tube and washed with WB. PBMCs were next centrifuged at 400× *g* for 10′ at RT with brakes on. The tube was decanted, flicked 3 times, and the PBMC pellet was resuspended in 50 mL WB. The tube was again centrifuged at 400× *g* for 10′ at RT, then resuspended in WB and counted before subsequent cryopreservation.

### 2.11. Peripheral Blood Mononuclear Cell Cryopreservation

A total of 10% dimethyl sulfoxide (DMSO, Sigma, Saint-Louis, MO, USA, cat#D2650-100ML) was mixed with 90% Heat-Inactivated Fetal Bovine Serum (Hi-FBS, Sigma, Saint-Louis, MO, USA, cat#F4135) to create cryopreservation solution (CPS) and cooled on wet ice, together with Nunc Cryotubes (Thermo Fisher, cat#377267), before start. Freshly isolated PBMCs were pelleted and resuspended in CPS and then aliquoted into cryovials at a final concentration of 1 × 10^7^ PBMCs/mL. Cryovials were then immediately transferred to a Nalgene Mr. Frosty cryo-freezing container (Thermo Fisher, Cat#5100-0001) and stored at −80 °C for 24 h before transfer to a liquid nitrogen (LN_2_) tank in vapor phase for longer term storage.

### 2.12. Peripheral Blood Mononuclear Cell Thawing

PBMCs of healthy donors were thawed at regular intervals to function as single compensation and FMO controls for patient whole blood. Thawing media consisted of RPMI Complete (RPMI1640 with 10% Hi-FBS with 200 IU Penicillin, 200 µg/mL Streptomycin and 2 mM L-Glutamine) and was pre-warmed before start. One cryovial was taken from LN_2_ storage and immediately transferred to a 37 °C water tank and thawed with regular flicking of the tube. The cryovial was transferred to a biosafety hood when only a small bit of ice remained, after which 1 mL of thawing media was added in a dropwise manner. The solution was then transferred to a 15-mL tube, and 10 mL of thawing media was slowly added. The tube was then centrifuged at 400× *g* for 10′ at RT. The pellet was resuspended in 15 mL of thawing media and again centrifuged at 400× *g* for 10′. The pellet was resuspended in 1 mL of running buffer, and thawed healthy donor PBMCs were then used for single compensation and FMO controls in flow cytometry analysis of the study participants’ samples.

### 2.13. Processing of Patient Whole Blood for Flow Cytometry Analysis

At the baseline, immediately pre-op, and POD1 timepoints, 8.0 mL of patient whole blood was collected in an EDTA tube via vein puncture and kept at RT. This tube was then immediately processed for flow cytometry analysis. Firstly, 2 mL of whole blood was transferred to a 50-mL tube with 25 mL of Ammonium-Chloride-Potassium (ACK) buffer pre-warmed to 37 °C to lyse erythrocytes. The tube was incubated for 12′ with tube inversion every 2′. ACK buffer was produced by mixing 150 mM NH_4_Cl (Sigma, cat# 254134), 10 mM KHCO_3_ (Sigma, cat# 237205), and 0.1 mM Na_2_EDTA (Sigma, cat# 324503) with 850 mL of H_2_O; then, pH was adjusted to 7.2–7.4 before achieving a final volume of 1000 mL. After 12′ incubation with ACK lysing buffer, the tube was filled to 50 mL with pre-warmed running buffer (0.05% bovine serum albumin in diH_2_O) and spun down at 400× *g* for 5′ at RT. The supernatant was then decanted, and the tube was flicked three times before resuspending the white blood cell (WBC) pellet in 50 mL running buffer. The tube was again spun down at 400× *g* for 5′ at RT before the supernatant was decanted and the pellet resuspended in 0.5 mL running buffer. To acquire a WBC-count, the sample was diluted 1:20 and loaded onto a hemocytometer. The four outer squares were counted and averaged. WBCs were counted twice, and the final count was averaged. A total of 1 × 10^6^ WBCs were then loaded per well on a 96-well round-bottom plate (Thermo Fisher, cat#475434) for further downstream flow cytometry processing.

### 2.14. Staining Procedure of WBC and PBMCs for Flow Cytometry

After patient blood was acquired, lysed, and resuspended as 2 × 10^6^ WBC/well in phosphate-buffered saline (PBS) in a 96-well round bottom plate, thawed PBMCs were added in the same plate with PBS (0.15 × 10^6^ PMBCs/well). Each plate was centrifuged at 400× *g* for 5′ at RT, decanted, and pellets were resuspended in Zombie live/dead dye (1:100, in PBS). Plates were incubated on wet ice in the dark for 15′, then washed with running buffer, and again centrifuged. Pellets were then resuspended in 50 µL 4% rat serum (in running buffer) and incubated for 10′ on wet ice in the dark. Antibody cocktails (all stained and FMO’s) were prepared, and 10 µL Brilliant Violet staining buffer was added (BD Biosciences, cat563794) together with running buffer, resulting in a final volume of 99 µL per cocktail. After incubation in rat serum, plates were centrifuged, decanted, and resuspended in antibody cocktail. Before incubation, P3 was added in a final concentration of 0.3 µM per well. Plates were then incubated for 20′ on ice in the dark. Wells were then washed with running buffer, centrifuged, and resuspended in 2% paraformaldehyde (diluted from 32% solution, Acros Organics, NJ, USA, cat#AC416785000) in running buffer and incubated for 20′ at RT in the dark. Plates were then transferred to 4 °C and run on the flow cytometer the same day or the next day.

### 2.15. Flow Cytometer Data Acquirement and Gating Strategy

Immunolabeled white blood cells were analyzed by flow cytometry using a BD LSR II Special Order Research Product (SORP) flow cytometer with BD High Throughput Sampler (HTS), running DiVa software version 8.01, and equipped with excitation laser lines at 488 nm (20 mW), 405 nm (50 mW), 594 nm (200 mW), and 355 nm (20 mW). The 594 nm line was operated at 125 mW.

Data analysis was performed in FlowJo software (BD Biosciences, Ashland, OR, USA), version 10. Cells were delineated using the following gating strategy to identify live single cells:

FSC-A versus SSC-A bivariate contour plots were used to initially gate on lymphocyte, monocyte, and granulocyte clusters. These populations were then visualized in an SSC-A versus Zombie Aqua fluorescence plot, with live cells gated as Zombie “negative”. The live cells were next shown in FSC-A versus FSC-H plots, and single cells were selected.

In the experiments involving patient samples, 10^6^ events, gated on live singlet populations, were collected for each stained experimental sample. A total of 10^5^ events were collected from above populations for each FMO, and 10,000 events were collected for samples stained with each individual antibody or dye for the purpose of compensation.

### 2.16. Luminex Assay

For protein isolation, Dulbecco’s phosphate-buffered saline with protease inhibitor cocktail (Roche Applied Science, Indianapolis, IN, USA) was added to each adipose tissue sample. Samples were then homogenized and centrifuged (2000× *g* × 5′) to remove debris, and then, the supernatant was centrifuged a second time (10,000× *g* × 10′). Supernatant was collected for quantitative protein analysis using Luminex multiple antigen magnetic bead assay (Luminex Corporation, Austin, TX, USA) according to the manufacturer’s instructions.

For analysis of serum samples, the following panel was used: neuronal growth factor, interleukin-6 (IL-6), insulin, leptin, interleukin-8 (IL-8), monocyte-chemoattractant protein-1 (MCP-1), tissue necrosis factor-alpha (TNF-α), interleukin-1β (IL-1β), adiponectin, lipocalin, resistin, adiposin, and plasminogen-activator inhibitor-1 (PAI-1). For analysis of adipose tissue samples, the following panel was used: NGF, IL-6, leptin, IL-8, hepatocyte growth factor (HGF), MCP-1, TNF-α, resistin, IL-1β, and PAI-1.

### 2.17. Statistical Analysis

Based on the goals of the study, the patient enrollment was not powered to test a specific mechanistic hypothesis or efficacy but rather to define infrastructure logistics, feasibility, and the general safety of outpatient PCR in vascular surgery patients needing an open operation.

Data are expressed as mean ± standard deviation (Mean ± SD). Statistical testing was conducted with Student’s *t*-tests and two-way ANOVA with Sidak’s multiple comparisons for continuous variables and Fisher’s exact test for categorical variables. Kaplan–Meier survival functions were generated and univariable Cox regression performed for time-to-event outcomes. Statistical analyses were performed with Graphpad (8.12) and R (4.0.5, R Foundation for Statistical Computing, Vienna, Austria).

## 3. Results

The study outline and design are depicted in Figure 1. From April 2019 until February 2020, 19 patients scheduled for elective vascular surgery consented to the study and were randomized. Out of 19 individuals, 12 were allocated to the PCR group and 7 patients to the AL group. Table 4 compares patient characteristics of both groups. Out of 12 PCR patients, 8 completed the study, while out of 7 AL patients, 3 completed the study. Table 5 summarizes the reasons for non-completion, which all occurred during the baseline stage of the study. Reasons for non-completion were either related to patient health (cardiac health, toxicology screening failure, emergency re-scheduling) or logistics (cancellation of surgery, missed baseline visit). Most importantly, none of the study participants reported issues with diet-compliance or tolerance, nor was there a diet-related withdrawal from the study.

Median follow-up was 504 days (IQR 385-715) using the reverse Kaplan–Meier technique, and follow-up at one year was 72.7%. No adverse events differed significantly between experimental groups in univariable Cox regression. Specifically, reintervention rates were similar between diet groups (HR 1.65, 95% CI 0.17–16.5, *p* = 0.67). In the PCR group, one patient suffered a myocardial infarction on POD3 after carotid endarterectomy, and one patient died due to a COVID infection one month after surgery. Supplemental Table S1 summarizes all AEs from date of index surgery.

**Figure 2:**
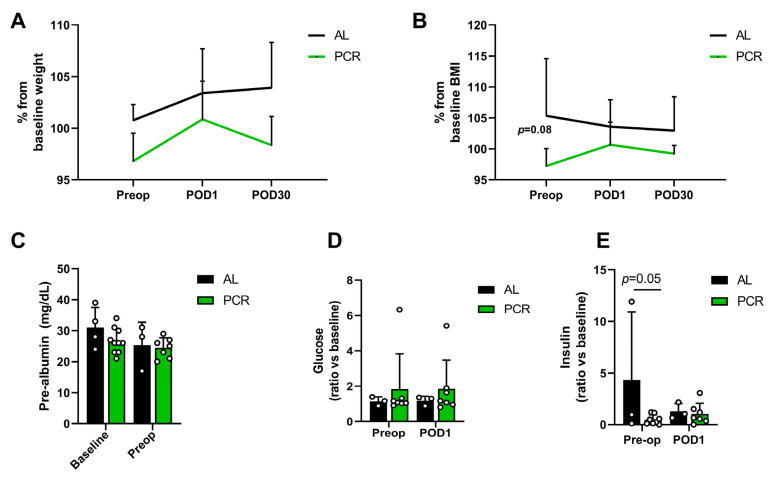
The effects of a short-term PCR on patient weight and glucose homeostasis. (**A**) Percent change in patient bodyweight at pre-op, POD1, and POD30 compared to weight at baseline visit. (**B**) Percent change in patient BMI at pre-op, POD1, and POD30 compared to BMI at baseline visit. (**C**) Pre-albumin levels (mg/dL) at baseline and pre-op in AL and PCR groups. (**D**) Patient blood glucose levels at pre-op and POD1, normalized to the respective patient’s baseline blood glucose level. (**E**) Patient serum insulin levels at pre-op and POD1, normalized to the respective patient’s baseline serum insulin level. Statistical testing was conducted via two-way ANOVA with Sidak’s multiple comparisons test, *n* = 3–10/group unless indicated otherwise.

Data collected via the MealLogger application allowed us to calculate that patients in the PCR group achieved a 29.4% calorie and 84.4% protein restriction on average compared to their pre-study intake. Table 6 lists average energy and protein intake per kilogram per day in both the AL and PCR group at baseline and during the period the PCR group was subjected to the dietary intervention. The PCR group appeared to have lost weight at the pre-operative visit (Figure 2A). The same trend was noted when comparing BMI at baseline with their pre-operative BMI (Figure 2B) in the PCR group. In terms of diet compliance, both the MealLogger data and weight parameters are indicative of adherence to the PCR diet. To test whether our PCR diet would result in malnutrition, we measured baseline and pre-op levels of pre-albumin, a short half-life protein [34]. Figure 2C shows no difference in pre-albumin levels between groups and before/after the diet, suggesting patients continued adequate nutrition during the PCR intervention. In our study, we were not able to detect a difference in baseline and pre-op glucose levels (Figure 2D). However, we did see a trend towards increased insulin sensitivity as a result of the PCR diet at the pre-operative timepoint as measured by insulin levels (Figure 2E).

**Figure 3:**
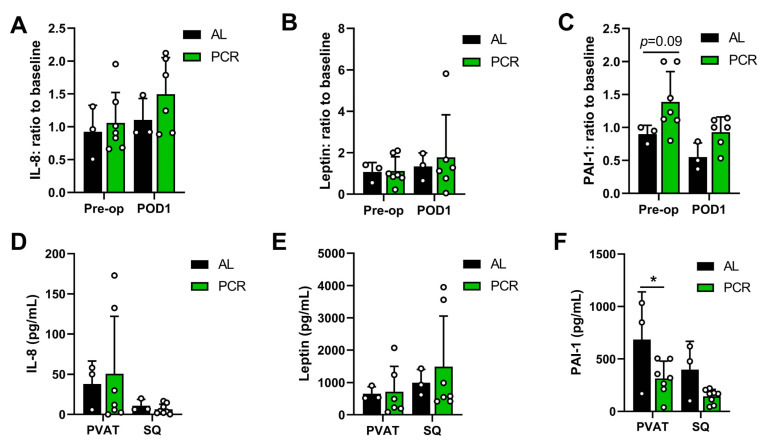
The effects of short-term PCR on cytokines and adipokines in circulation and in subcutaneous and perivascular adipose depots. (**A**–**C**) Cytokines and adipokines in serum of AL/PCR patients, baseline versus pre-op in POD1 ratio. (**A**) Interleukin-8 (IL-8). (**B**) Leptin. (**C**) Plasminogen activator inhibitor-1 (PAI-1). (**D**,**E**) Cytokines and adipokines in perivascular (PVAT) and subcutaneous (SQ) adipose tissue of AL/PCR patients. (**D**) IL-8 in pg/mL. (**E**) Leptin in pg/mL. (**F**) PAI-1 in pg/mL. Statistical testing was conducted via two-way ANOVA with Sidak’s multiple comparisons test, *n* = 3–7/group unless indicated otherwise. * <0.05.

We also investigated circulating and local cytokine and adipokine regulation pre- and post-surgery in response to the diet, as DR is known to reduce inflammation. We did not detect changes in any of the circulating cytokines (NGF, IL-6, IL-8, MCP-1, TNF-α, or IL-1β; Figure 3A, IL-8 shown as an example) or adipokines (adiponectin, lipocalin, resistin, adiposin, or leptin; Figure 3B, leptin shown as an example) measured in serum at baseline, pre-operatively, and post-op. However, plasminogen activator inhibitor-1 (PAI-1) appeared to be increased in pre-operative PCR patients compared to baseline levels (Figure 3C). Additionally, we investigated regulation of these same markers at the perioperative timepoint in both subcutaneous and perivascular (PVAT) adipose tissue. We detected no apparent change in levels of the aforementioned cytokines and adipokines (Figure 3D,E). Interestingly, PAI-1 was significantly downregulated in the PVAT of PCR patients compared to patients who were AL fed (Figure 3F).

We assayed circulating leukocyte subsets at baseline, pre-operative, and post-operative timepoints in combination with a hydrogen sulfide (H_2_S) probe that detects intracellular levels of H_2_S [33]. Supplemental Figure S1 highlights the gating strategy employed to interrogate the different leukocyte subsets, based on a previously published strategy. [32] None of the interrogated cell populations (granulocytes, monocyte subtypes, T-cell subsets, NK cells, dendritic cells, and B cells) showed any differences between diet groups and before/after surgery (Supplemental Figure S2).

**Figure 4:**
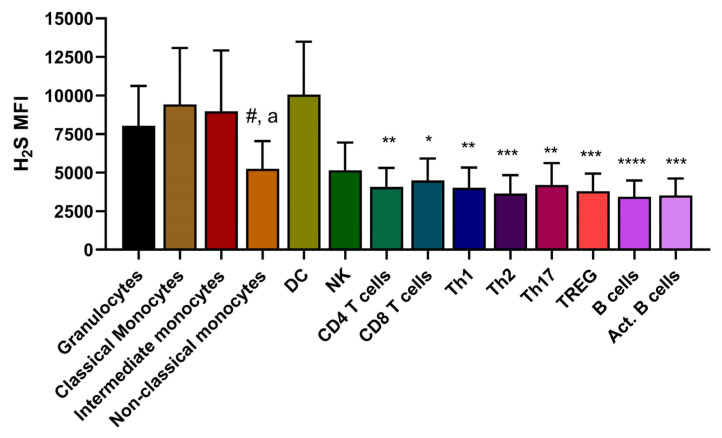
Levels of endogenous intra-cellular H_2_S differ between human innate and adaptive immune cells. In the PCR group, all cell subtypes were compared at baseline for their intra-cellular levels of H_2_S. Unless indicated otherwise, the differences between innate and adaptive cell types were highlighted via comparisons between granulocytes and each cell subset. Statistical testing was conducted via one-way ANOVA with Sidak’s multiple comparisons test, *n* = 6/group. * <0.05, ** <0.01, *** <0.001, **** <0.0001. # = classical monocytes vs non-classical monocytes, *p* =< 0.01. a = intermediate monocytes vs non-classical monocytes, *p* < 0.05.

Lastly, we measured intracellular H_2_S in each separate leukocyte subset. Although there was no detectable difference in H_2_S levels between diet groups and timepoints, we were able to make some observations. Intriguingly, cells considered part of the innate immune system consistently had higher levels of intra-cellular H_2_S compared to cells of the adaptive immune system (Figure 4). Secondly, although classical pro-inflammatory monocytes possess high levels of H_2_S, their non-classical anti-inflammatory counter parts have significantly lower levels of H_2_S (Figure 4), perhaps suggestive of a role for H_2_S in mediating the inflammatory state of this cell type.

## 4. Discussion

Here, we present the results of a randomized controlled trial in outpatient pre-operative dietary restricted versus ad-libitum-fed patients scheduled for vascular surgery. This work expands on our previously published pilot study assessing the safety of such a short-term pre-operative PCR in inpatient vascular surgery patients [30]. Although the runtime of the trial and enrollment was shortened substantially due to the COVID-19 pandemic, we were still able to consent 19 individuals in 10 months. Ultimately, 11 patients completed the study, and we observed no diet-related reasons for withdrawal from the study once enrolled. The dietary intervention was well tolerated, with none of the study participants reporting deviation from their PCR diet. The eight patients enrolled in the PCR arm achieved 29.4% calorie and 84.4% protein restriction on average, and these findings were supported by a decrease in weight and BMI in the PCR group compared to their baseline levels. There was no detectable difference in occurrence of adverse events between diet groups, although this trial was not designed to test efficacy but rather feasibility of this diet. Furthermore, nor were there any hypoglycemic or hyperglycemic incidents in the PCR group. Circulating leukocyte cell populations were maintained under PCR. Overall, these results indicate that short-term PCR in vascular surgery patients appears both feasible and safe.

Perioperative glucose regulation has been linked to improved outcomes and impaired wound healing in several studies [35,36]. In a previous preclinical study, our group was able to link short-term pre-operative protein restriction with improved glucose homeostasis both pre- and post-operatively [21]. In the current study, we were not able to detect any differences in pre- and post-op glucose levels. However, we did find lower pre-operative circulating insulin levels, suggestive of possible effect of PCR on glucose homeostasis/insulin signaling. A future, larger scale trial should be able to delineate whether short-term PCR improves glucose metabolism and whether this can be linked to improved clinical outcomes and wound healing.

Intriguingly, PAI-1 levels were lower in the PVAT of patients after four days of PCR. Several studies have implicated adipocytes as the main producers of PAI-1, and production of PAI-1 in adipocytes is triggered by hyperglycemia and increased insulin resistance [37]. Indeed, PAI-1 is elevated in patients with diabetes type 2 compared to lean control subjects [38], but elevated levels of PAI-1 are also implicated in (components of) cardiovascular disease [37], including vascular inflammation and atherosclerosis. [39] Our current study is not adequately powered to truly establish a possible association between PAI-I, PVAT, and PCR; therefore, future adequately powered studies should investigate a possible association between PCR and regulation of PAI-I in adipose tissue in the perioperative period and explore links between PAI-I and potential functional benefits of PCR.

We did not observe any major perturbations in the circulating leukocyte subsets with our PCR intervention, suggesting that these highly evolved cells will be available to participate in the physiologic response to surgical trauma. However, our small sample size limited the conclusions we could draw from our analysis of the innate and adaptive immune system. A larger scale trial that includes the same leukocyte flow cytometry panel should yield an answer to the question of involvement of the immune system in any potential benefit of the PCR diet.

Previous studies by our group have explicitly linked pre-operative DR with upregulation of endogenous H_2_S in endothelial cells [16,22,23]. Despite not detecting any upregulation in endogenous H_2_S in immune cells as a result of diet or surgery, we did, however, detect remarkable differences in endogenous levels of intra-cellular H_2_S between innate and adaptive immune cells, with higher levels in innate cells. Within the innate immune cell groups, there also were strikingly lower H_2_S levels in anti-inflammatory monocytes compared to their pro-inflammatory counter parts. Both observations, to our knowledge, grant a first look into endogenous H_2_S immune cell biology since current knowledge is based on the interaction between exogenous H_2_S and innate or adaptive immune cells [40]. Whether specific levels of endogenous H_2_S can be directly linked to an immune cells’ inherent inflammatory state requires further investigation.

## 5. Conclusions

The present study provides direct evidence that highlights the safety and feasibility of short-term pre-operative dietary restriction in patients scheduled for elective surgery. Scheduling and logistical issues can make dietary intervention before elective surgery challenging, but PCR is not untenable. As shown here and previously, both in- and out-patient PCR interventions are feasible and safe, with outpatient trials being a more translational and sustainable intervention as patients incorporate this into their daily lives. In addition to the growing body of preclinical and clinical studies on vascular surgery patients, several other studies have shown feasibility and safety of DR in coronary bypass surgery [41] and patients scheduled for liver resection [42]. Pre-operative PCR specifically performed in living kidney donors in order to enhance recipient kidney function has previously been shown to be safe and feasible [43]. More recently, a follow-up study pointed towards improved kidney function after transplantation [44]. Excitingly, this implicates a role for PCR beyond vascular surgery, which could potentially improve patient outcomes across many fields, resulting in better healthcare outcomes and lower costs for patients and hospitals. Future studies should expand upon these in terms of patient recruitment and multi-center trials as well as by working to validate other preclinical observations, such as improved glucose homeostasis [21], wound healing [21], and improved vascular reconstruction durability [22].

## Figures and Tables

**Table 1:** Scandishake macronutrient ratio in Kcal and %.

Macronutrients	Kcal	%
Protein	19.64	4
Carbohydrates	255.32	42
Fat	216.04	44
Total	491	100

**Table 2:** Antibody cocktail for the study.

Antibody (Mouse α Human)	Important Marker for	Fluorophore	Clone	Manufacturer	Catalog Number	Final Volume (in 100 µL)
CD3	T cells	AF-700	UCHT1	Invitrogen	56-0038-42	5 µL
CD4	CD4 T cells	PercP-Cy5.5	RPA-T4	BD Biosciences	560650	1.25 µL
CD8a	CD8 T cells	BV785	RPA-T8	Biolegend	301046	5 µL
CD14	Monocytes	PE-Cy7	M5E2	BD Biosciences	560919	5 µL
CD16	Monocytes	PE-CF594	3G8	Biolegend	302054	2.5 µL
CD19	B cells	BV650	HIB19	Biolegend	302238	10 µL
CD25	Treg cells	APC	M-A251	BD Biosciences	590987	20 µL
CD38	T cell/B cell	AF 488	HIT2	Biolegend	303512	2.5 µL
CD56	B cells	BV605	HCD56	Biolegend	318334	5 µL
CD127	Treg cells	BV711	A019D5	Biolegend	351328	5 µL
CD183 (CXCR3)	Th1 cells	PE	11A9	BD Biosciences	743356	10 µL
CD196 (CCR6)	Th1, Th2, Th17	BUV395	1C6/CXCR3	BD Biosciences	560928	1.25 µL
HLA-DR	Dendritic cells	PE	LN3	Invitrogen	47-9956-42	0.625 µL

**Table 3:** CD4 antibodies.

Antibody (Mouse α Human)	Fluorophore	Clone	Manufacturer	Catalog Number	Final Volume (in 100 µL)
CD4	APC	RPA-T4	BD Biosciences	561840	20 µL
CD4	BUV395	RPA-T4	BD Biosciences	564724	1.25 µL
CD4	PE	RPA-T4	BD Biosciences	561843	20 µL
CD4	BV605	RPA-T4	Biolegend	300556	2.5 µL
CD4	AF488	RPA-T4	Biolegend	300519	5 µL
CD4	BV711	RPA-T4	Biolegend	300558	2.5 µL

**Table 4:** Baseline patient characteristics, post-randomization. Age-differences between groups was tested via Student’s *t*-test. Between-group differences of all other patient characteristics were tested via Fisher’s exact test.

Baseline Characteristics	AL (4)	PCR (12)	Statistical Difference
Age in years (SD)	66.5 (11.3)	64.3 (12.8)	*p* = 0.75
Gender Male (total)	2 (4)	8 (12)	
Female (total)	2 (4)	4 (12)	*p* = 0.54
Smoking (total)	1 (4)	4 (12)	*p* = 0.83
Diabetes (total)	2 (4)	4 (12)	*p* = 0.54
Hypertension (total)	4 (4)	9 (12)	*p* = 0.51
Hypercholesterolemia (total)	1 (4)	4 (12)	*p* > 0.99
History of Malignancy (total)	0 (4)	2 (12)	*p* > 0.99
Transient ischemic attack/Stroke (total)	1 (4)	2 (12)	*p* > 0.99
Cardiovascular disease	2 (4)	2 (12)	*p* = 0.22
Peripheral vascular disease	2 (4)	7 (12)	*p* > 0.99
Renal insufficiency	1 (4)	2 (12)	*p* = 0.53

**Table 5:** Reasons for non-completion after initial consent and randomization. Transcarotid artery revascularization (TCAR).

Reasons for Non-Completion of the Study	*AL* *(7)*	*PCR* *(12)*
Procedure changed to TCAR (outside of protocol at the time)	1	
Failed toxicity screening	1	
Patient failed to show for baseline, did not want to reschedule		1
	1	
Surgery cancelled		1
Failed pre-operative cardiac clearance	1	1
Surgery rescheduled emergently	1	
Patient opted out after baseline (did not want to deal with research, food diary, etc. before surgery)		1
Remaining study participants who completed the trial	3	8

**Table 6:** Energy and protein intake in AL and PCR groups.

	PCR	AL
Baseline energy intake	23.04 Kcal/kg/day	11.97 Kcal/kg/day
Intervention energy intake	15.4 Kcal/kg/day	20.08 Kcal/kg/day
Baseline protein intake	1.06 g/kg/day	0.34 g/kg/day
Intervention protein intake	0.16 g/kg/day	0.76 g/kg/day

## Data Availability

Raw data sets will be made available upon request.

## Supplementary Materials

The following are available online at https://www.mdpi.com/article/10.3390/nu13114024/s1, Figure S1: Global gating strategy for leukocyte subsets. (**A**) Via SSC-A and FSC-A, granulocyte and lymphocytes/monocytes populations are defined, followed by dead cells exclusion via zombie dye. Via FSC-A and FSC-H, single cells are identified. (**B**) CD3 and CD19 markers allow for discrimination of T cells (CD3+ CD19−) and B cells (CD3− CD19+). Within the T-cell population, CD4+ and CD8+ T cells, regulatory T cells (CD4+ CD25+ CD127−), and, e.g., Th17 cells (CXCR3− CCR6+), can be identified. The MFI for P3 of NK cells has been plotted as an example as well as CD4+ (red) and CD8+ (blue) T cells. (**C**) In CD3− CD19- cells, classical (CD14+ CD16−), intermediate (CD14+ CD16+), and non-classical (CD14− CD16+) monocytes can be identified. In addition, the MFI for P3 of the classical (red), intermediate (green), and non-classical (blue) monocytes is shown. (**D**) Gating strategy to identify granulocytes and granulocyte P3 MFI. (**E**) P3 MFI of T cells (blue) and classical monocytes (red); Figure S2: Different subsets of leukocytes at baseline, pre-op and post-op day 1 timepoints. All subsets are graphed as a percentage of their parent cell population: (**A**) Granulocytes. (**B**) Lymphocytes. (**C**) CD4 T cells. (**D**) CD8 T cells. (**E**) Th1 memory cells. (**F**) Th2 memory cells. (**G**) Th17 cells. (**H**) T-regulatory cells. (**I**) Intermediate monocytes. (**J**) Classically activated monocytes. (**K**) Non-classical activated monocytes. (**L**) Natural Killer cells (NK). (**M**) Dendritic cells (DCs). (**N**) B cells. (**O**) Activated B cells; Table S1: Adverse events (AE) at 30 days and one year of follow-up.
