# Supplementary material for: Short-Term Pre-Operative Protein Caloric Restriction in Elective Vascular Surgery Patients: A Randomized Clinical Trial
Source: Nutrients. 2021 Nov 11;13(11):4024. doi: 10.3390/nu13114024 (PMC8621550; doi:10.3390/nu13114024)
Supplement: Supplementary file 1 [file nutrients-13-04024-s001.zip › nutrients-1439348-supplementary.pdf]

Table S1. Adverse events (AE) at 30 days and one year of follow-up.

Event	30 Days			1 Year [a]	
	AL, N = 3[b]	PCR, N = 8[b]	*p*-Value[c]	AL, N = 3 [b]	PCR, N = 8 [b]
Death (All Cause)	0 (0%)	0 (0%)		0 (0%)	1 (12%)
Death (Cardiovascular)	0 (0%)	0 (0%)		0 (0%)	0 (0%)
Stroke	0 (0%)	0 (0%)		0 (0%)	0 (0%)
Myocardial Infarction	0 (0%)	1 (12%)	>0.9	0 (0%)	1 (12%)
Coronary Revasc.	0 (0%)	0 (0%)		0 (0%)	0 (0%)
Conduit Thrombosis	0 (0%)	2 (25%)	>0.9	0 (0%)	2 (25%)
Reintervention	0 (0%)	3 (38%)	0.5	1 (33%)	3 (38%)

[a] 1 year counts inclusive of 30 day counts; [b] n (%); [c] Fisher's exact test.

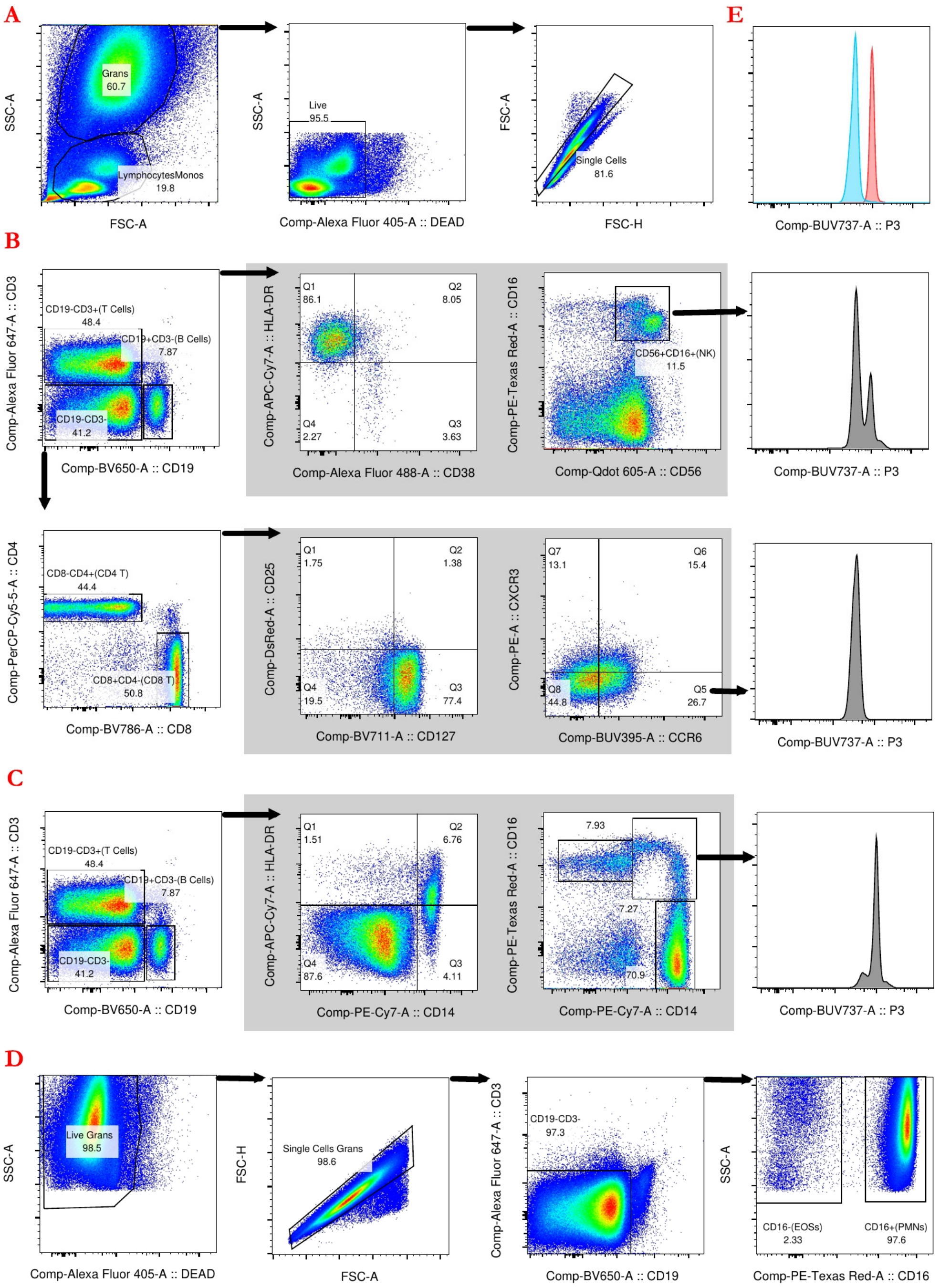

Figure S1. Global gating strategy for leukocyte subsets. (**A**) Via SSC-A and FSC-A, granulocyte and lymphocytes/monocytes populations are defined, followed by dead cells exclusion via zombie dye. Via FSC-A and FSC-H, single cells are identified. (**B**) CD3 and CD19 markers allow for discrimination of T cells (CD3+ CD19−) and B cells (CD3− CD19+). Within the T-cell population, CD4+ and CD8+ T cells, regulatory T cells (CD4+ CD25+ CD127−), and, e.g., Th17 cells (CXCR3− CCR6+), can be identified. The MFI for P3 of NK cells has been plotted as an example as well as CD4+ (red) and CD8+ (blue) T cells. (**C**) In CD3− CD19- cells, classical (CD14+ CD16−), intermediate (CD14+ CD16+), and non-classical (CD14− CD16+) monocytes can be identified. In addition, the MFI for P3 of the classical (red), intermediate (green), and non-classical (blue) monocytes is shown. (**D**) Gating strategy to identify granulocytes and granulocyte P3 MFI. (**E**) P3 MFI of T cells (blue) and classical monocytes (red).

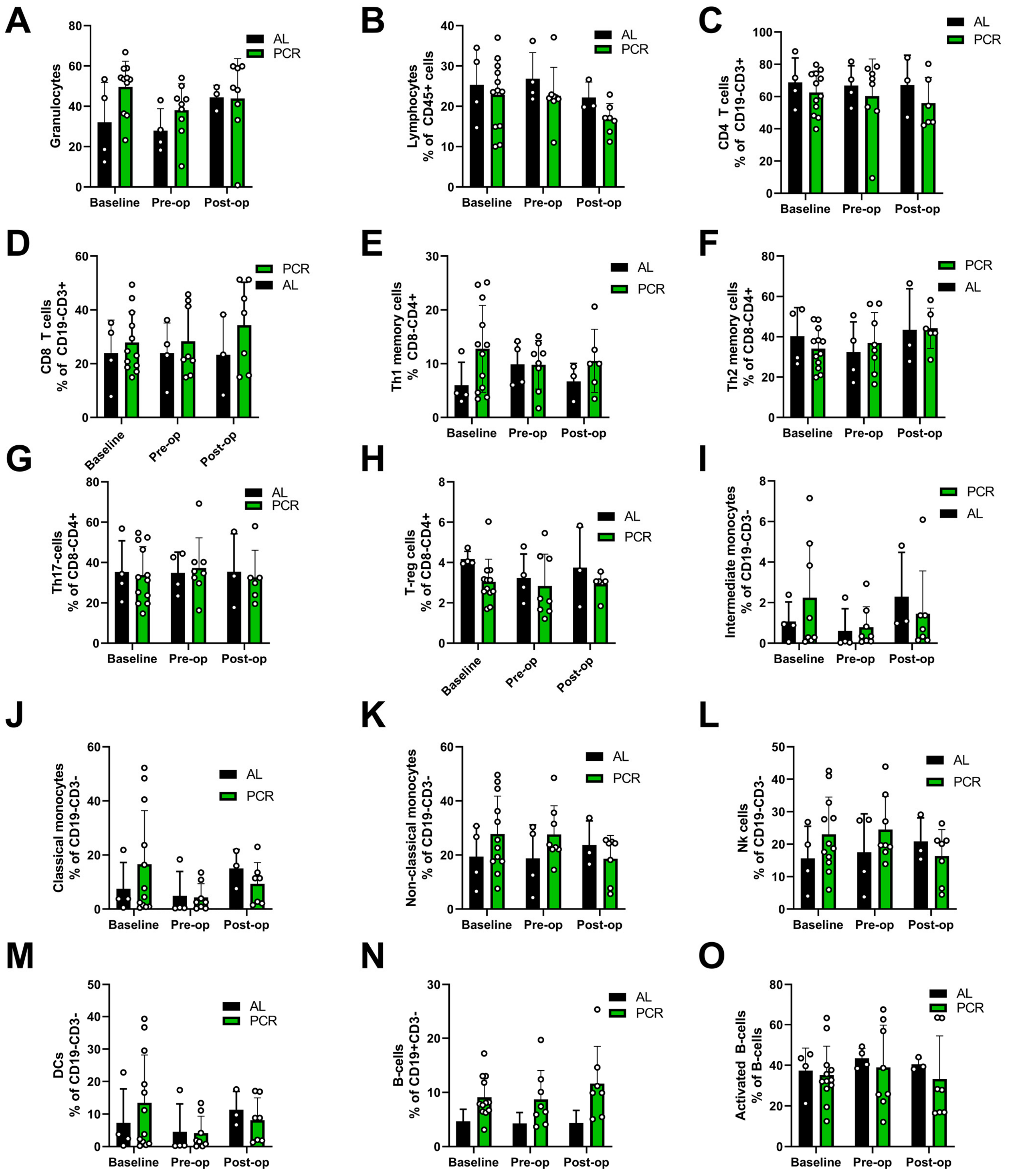

Figure S2. *Different subsets of leukocytes at baseline, pre-op and post-op day 1 timepoints.* All subsets are graphed as a percentage of their parent cell population: (**A**) Granulocytes. (**B**) Lymphocytes. (**C**) CD4 T cells. (**D**) CD8 T cells. (**E**):Th1 memory cells. (**F**) Th2 memory cells. (**G**) Th17 cells. (**H**) T-regulatory cells. (**I**) Intermediate monocytes. (**J**) Classically activated monocytes. (**K**) Non-classical activated monocytes. (**L**) Natural Killer cells (NK). (**M**) Dendritic cells (DCs). (**N**) B cells. (**O**) Activated B cells.
